# Myeloid-derived suppressor cells in non-neoplastic inflamed organs

**DOI:** 10.1186/s41232-018-0076-7

**Published:** 2018-09-17

**Authors:** Sho Sendo, Jun Saegusa, Akio Morinobu

**Affiliations:** 1 Division of Rheumatology and Clinical Immunology, Department of Internal Medicine, Kobe University Graduate School of Medicine, 7-5-2 Kusunoki-cho, Chuo-ku, Kobe, 650-0017 Japan; 2 Division of Laboratory Medicine, Kobe University Graduate School of Medicine, 7-5-2 Kusunoki-cho, Chuo-ku, Kobe, 650-0017 Japan

**Keywords:** MDSC, Inflammation, T cell, Myeloid cell, Organ, Plasticity

## Abstract

**Background:**

Myeloid-derived suppressor cells (MDSCs) are a highly heterogeneous population of immature myeloid cells with immunosuppressive function. Although their function in tumor-bearing conditions is well studied, less is known about the role of MDSCs in various organs under non-neoplastic inflammatory conditions.

**Main body:**

MDSCs are divided into two subpopulations, G-MDSCs and M-MDSCs, and their distribution varies between organs. MDSCs negatively control inflammation in inflamed organs such as the lungs, joints, liver, kidneys, intestines, central nervous system (CNS), and eyes by suppressing T cells and myeloid cells. MDSCs also regulate fibrosis in the lungs, liver, and kidneys and help repair CNS injuries. MDSCs in organs are plastic and can differentiate into osteoclasts and tolerogenic dendritic cells according to the microenvironment under non-neoplastic inflammatory conditions.

**Conclusion:**

This article summarizes recent findings about MDSCs under inflammatory conditions, especially with respect to their function and differentiation in specific organs.

## Background

Myeloid-derived suppressor cells (MDSCs), which are best known for their role in tumor-bearing conditions, also mediate the resolution of inflammatory diseases. MDSCs can play conflicting roles in inflammation because the phenotypes and functions of this plastic, highly heterogeneous population depend largely on the local microenvironment. We here discuss the role of MDSCs under non-neoplastic inflammatory conditions, particularly in individual organs.

## Main text

### What are MDSCs?

MDSCs are a heterogeneous population of immature myeloid cells with immunosuppressive function. MDSCs were initially described in 1987 in a mouse model of lung cancer [1]. More recently, roles for MDSCs have been implicated in non-neoplastic inflammatory conditions such as infection, allergy, and autoimmune diseases [2, 3]. The major populations of bone marrow-derived myeloid cells are neutrophils, monocytes, macrophages, and dendritic cells (DCs), which are differentiated from hematopoietic progenitor cells via granulocyte-macrophage progenitors (GMPs). MDSCs are also differentiated from the common progenitors especially under pathogenic conditions [4]. Strong but short activation signals coming from acute infection or trauma induce classic myeloid cell activation, e.g., neutrophil, monocyte/macrophage, and DC activation. On the other hand, weak but chronic activation signals from malignancies, chronic infections, and inflammatory diseases favor the development of MDSC [4].

MDSCs are classified as granulocytic (G)-MDSCs or monocytic (M)-MDSCs. G-MDSCs are morphologically similar to neutrophils, while M-MDSCs are morphologically similar to monocytes. These subtypes are distinguished from neutrophils or monocytes by their morphology, surface phenotype, and function (Table 1). In mice, MDSCs are phenotypically defined as CD11b^+^Gr1^+^ cells, which are further classified into G-MDSCs as CD11b^+^Ly6G^+^Ly6C^low^ cells and M-MDSCs as CD11b^+^Ly6G^−^Ly6C^hi^ cells [5]. In humans, markers for the entire MDSC population have not been clearly determined. Among peripheral blood mononuclear cells (PBMCs), the equivalent to G-MDSCs is defined as CD11b^+^CD14^−^CD15^+^ (or CD66b^+^) cells, and that to M-MDSCs as CD11b^+^CD14^+^HLA-DR^−/low^CD15^−^ cells [6]. As it is difficult to phenotypically distinguish G-MDSCs from neutrophils, or M-MDSCs from monocytes, it is important to prove whether or not these cells have suppressive function. While G-MDSCs and M-MDSCs are differentiated from common progenitors, e.g., GMPs [4], only immature neutrophils and monocytes can be converted to G-MDSCs and M-MDSCs, respectively [7].

**Table 1:** Comparisons of morphology, phenotype, and function between myeloid-derived cells

	Morphology	Surface phenotype	Immune suppression
Mouse			
Neutrophils	Round shape with a segmented nucleus	CD11b^+^Ly6G^hi^Ly6C^lo^	−
Monocytes	Round shape with a indented nucleus	CD11b^+^Ly6G^−^Ly6C^hi^	−
Macrophages	Round shape with pseudopodia	CD11b^+^F4/80^hi^Ly6G^−^Ly6C^lo^	−
Dendritic cells	Dendritic shape with polypodia	CD11b^+^CD11c^+^Ly6G^−^Ly6C^−/lo^ (classical)	−
		CD11b^−^CD11c^+^Ly6G^−^Ly6C^−^ (classical)	−
		CD11b^−^CD11c^lo^Ly6G^−^Ly6C^+^PDCA-1^+^ (plasmacytoid)	−
Fibrocytes	Spindle shape	CD11b^+^ColI^+^Ly6G^−^Ly6C^+^	−
G-MDSCs	Round shape with a banded nucleus	CD11b^+^Ly6G^+^Ly6C^lo^	+
M-MDSCs	Round shape with a indented nucleus	CD11b^+^Ly6G^−^Ly6C^hi^	++
Human			
Neutrophils	Round shape with a segmented nucleus	CD11b^+^CD14^−^CD15^+^CD66b^+^LOX-1^−^	−
Monocytes	Round shape with a indented nucleus	CD14^+^CD15^−^CD16^−^HLA-DR^+^ (classical)	−
		CD14^+^CD15^−^CD16^+^HLA-DR^+^ (intermediate)	−
		CD14^−^CD15^−^CD16^+^HLA-DR^+^ (non-classical)	−
Macrophages	Round shape with pseudopodia	CD14^+^CD15^−^CD16^+^CD80^+^HLA-DR^+^ (M1)	−
		CD11b^+^CD14^+^CD15^−^CD206^+^CD163^+^HLA-DR^+^ (M2)	±
Dendritic cells	Dendritic shape with polypodia	CD14^−^CD16^−^CD1C^+^ (classical)	−
		CD14^−^CD16^−^CD141^+^ (classical)	−
		CD14^−^CD16^−^CD303^+^ (plasmacytoid)	–
Fibrocytes	Spindle shape	CD11b^+^ColI^+^CD13^+^CD34^+^CD45RO^+^HLA-DR^+^	−
G-MDSCs	Round shape with a annular nucleus	CD11b^+^CD14^−^CD15^+^CD66b^+^LOX-1^+^	+
M-MDSCs	Round shape with a indented nucleus	CD14^+^CD15^−^HLA-DR^−/lo^	++

*Abbreviations*: *HLA* human leukocyte antigen, *Lox-1* lectin-type oxidized LDL receptor 1, *PDCA-1* plasmacytoid dendritic cell antigen-1

Macrophages are also known to be a highly heterogeneous population and M1/M2 subsets of macrophages are widely accepted [8]. Macrophages are differentiated from GMPs via monocyte-DC precursors and Ly6C^hi^ monocytes [9]. Although M2 macrophages and M-MDSCs have a common origin and work as anti-inflammatory cells, these cells differ in several respects. For example, M-MDSCs express lower levels of MHC-II than M2 macrophages do, indicating that M-MDSCs are more immature populations. In addition, M-MDSCs, but not M2 macrophages, produce inducible nitric oxide synthase (iNOS), whereas arginase-I (Arg-I) is produced in both cell types [10].

MDSCs are thought to suppress immune function by depleting lymphocytes’ nutrients, generating oxidative stress, and inducing regulatory T cells (Tregs) [11]. Signal transducer and activator of transcription (STAT), such as STAT1 and STAT3, is involved in regulating MDSC function. IFN-γ and interleukin (IL)-1β can trigger STAT1 signaling, leading to high levels of iNOS and Arg-I [12]. The resulting l-arginine depletion and nitric oxide (NO) production suppresses T cells. A lack of l-arginine decreases ζ-chain expression in the T cell receptor complex and arrests the proliferation of antigen-activated T cells [13]. On the other hand, NO production abolishes T cell function by inhibiting MHC-II expression and inducing T cell apoptosis [14]. Although both MDSC subsets can increase Arg-I levels, M-MDSCs produce higher levels of NO than do G-MDSCs [15]. T cell function is also regulated by modulating the CD3 ζ-chain expression, which is controlled by reactive oxygen species (ROS) production [16]. ROS are secreted primarily by G-MDSCs, and this secretion is upregulated by increased STAT-3 activation by IL-6, IL-10, and GM-CSF [11]. M-MDSCs, but not G-MDSCs, can promote Treg induction from purified CD4^+^ T cells [17], and Tregs suppress the activation and expansion of autoreactive T cells [18].

### MDSC function in inflamed organs

This section describes the latest studies concerning the roles of MDSCs in individual organs, in both humans and mouse disease model systems (Table 2).

**Table 2:** The roles of MDSCs in individual organs

Organ	Disease	Species	Surface phenotype	MDSC function	Reference
Lung	Asthma	Mouse (HDM)	CD11b^+^Gr1^int^F4/80^+^	Suppression of Th2 cell reactivation	[16]
		Human	CD11b^+^CD14^+^CD16^−^HLA-DR^−^ (BAL)	NA	[19]
	COPD	Human	Lineage^−^HLA-DR^−^CD33^+^CD11b^+^ (peripheral blood)	NA	[20]
			Collagen type 1^+^CD45^dim^CD34^−^CD1^−^CD15^+^ (lung, peripheral blood)	NA	[21]
	Interstitial lung disease	Mouse (SKG)	CD11b^+^Gr1^+^ (lung)	Suppression of T cell proliferation	[22]
	Lung fibrosis	Mouse (silica)	CD11b^+^Ly6C^+^CCR2^+^ (lung)	Suppression of T cell proliferation, promotion of lung fibrosis by producing TGF-β1	[23]
	Pulmonary hypertension	Human	CD11b^+^CD33^+^MHC-II^−^ (peripheral blood)	NA	[24]
Joint	Rheumatoid arthritis model	Mouse (PGIA)	CD11b^+^Ly6G^hi^Ly6C^int/lo^ (synovial fluid)	Suppression of DC maturation and T cell proliferation	[27]
		Mouse (CIA)	CD11b^+^Gr1^+^ (spleen)	Suppression of T cell proliferation and Th17 cell differentiation, amelioration of arthritis	[28–30]
		Mouse (SKG)	CD11b^+^Gr1^+^ (spleen, BM)	Suppression of T cell proliferation, amelioration of arthritis	[31]
	Rheumatoid arthritis	Human	CD11b^+^CD33^+^HLA-DR^lo/−^CD14^−^CD15^+^ (synovial fluid)	NA	[33]
Liver	Immune-mediated hepatitis	Mouse (TGFb1−/−)	CD11b^+^Ly6G^lo^Ly6C^hi^	Suppression of CD4^+^ T cell proliferation	[42, 43]
		Mouse (Con A)	CD11b^+^Ly6G^+^Ly6C^lo^		
	Fulminant hepatitis	Mouse (D-Gal/LPS)	CD11b^+^Ly6G^lo^Ly6C^hi^	Suppression of CD4^+^ T cell proliferation and cytokine production	[44]
			CD11b^+^Ly6G^hi^Ly6C^int^		
	Liver fibrosis	Mouse (carbon tetrachloride)	CD11b^+^Ly6G^−^Ly6C^hi^F4/80^+^	Amelioration of fibrosis through inhibition of hepatic stellate cells	[45]
			CD11b^+^Ly6G^+^Ly6C^lo^F4/80^−^		
		Mouse (bile duct ligation)	CD11b^+^Ly6C^+^		[46]
	HCV hepatitis	Human	CD11b^+^ HLA-DR^lo^ CD33^+^ CD14^+^	Inhibition of T cell proliferation and IFN-γ production	[40, 41]
			CD11b^+/lo^ HLA-DR^lo/−^ CD33^+^ CD14^+^		
Kidney	AKI	Mouse (ischemia-reperfusion)	CD11b^+^Ly-6G^+^Ly-6C^low^ (kidney)	Attenuation of AKI via suppression of T cell infiltration, downregulation of pro-inflammatory cytokines	[50]
	FSGS	Mouse (doxorubicin)	CD11b^+^Gr1^+^ (peripheral blood, BM, spleen, kidney-draining lymph nodes, and kidney)	Attenuation of renal injury via inducing regulatory T cells	[51]
		Human	CD11b^+^HLA-DR^−^CD14^−^CD15^+^ (peripheral blood)	Suppression of T cell proliferation	[51]
	Kidney fibrosis	Mouse (adenine-enriched diet)	CD11b^+^Ly6G^+^ (kidney)	Suppression of T cell proliferation and kidney fibrosis	[46]
Intestine	IBD	Mouse (VILLIN-hemagglutinin)	CD11b^+^Gr1^+^ (spleen, intestine)	Suppression of CD8^+^ T cell proliferation via NO production	[54]
		Mouse (TNBS)	CD11b^+^Ly-6G^+^Ly-6C^lo^ (BM)	Attenuation of colitis via suppression of MPO activity and serum IL-6 levels	[55]
		Mouse (DSS)	CD11b^+^Gr1^+^ (spleen, BM)	Attenuation of colitis via suppression macrophages in the lamina propria	[56]
		Human	CD14^+^HLA-DR^−/lo^ (peripheral blood)	Suppression of PBMC proliferation and IFN-γ production	[54]
CNS	Multiple sclerosis	Mouse (EAE)	CD11b^+^Ly-6C^hi^ (peripheral blood, BM, spleen, and CNS)	Suppression of CD4 and CD8 T cell proliferation via NO production, Enhancement of T cell apoptosis and attenuation of EAE	[61]
		Mouse (EAE)	CD11b^+^Ly6G^+^ (peripheral lymphoid compartment, CNS)	Attenuation of EAE via inhibition of encephalitogenic Th1 and Th17 immune responses.	[63]
		Human	HLA-DR^−/lo^CD14^−^CD33^+^CD15^+^ (peripheral blood)	Suppression of autologous CD4^+^ T cell activation and proliferation	[63]
Eye	Spinal cord injury (SCI)	Mouse	CD11b^+^Ly6C^+^Ly6G^−^ (spinal cord)	Promoting the repair process after SCI	[64]
	Stroke	Mouse	CD11b^+^Ly6C^+^MHC-II^lo^ (spleen)	Suppression of T cell proliferation	[65]
		Human	CD11b^+^CD33^+^HLA-DR^−^ (peripheral blood)	NA	[65]
	Uveoretinitis	Mouse (EAU)	CD11b^+^Ly6G^−^Ly6C^+^ (peripheral blood, spleen, retina)	Suppression of T cell proliferation, attenuating uveoretinitis	[67, 68]
		Human (posterior uveitis)	HLA-DR^−^CD11b^+^CD33^+^	NA	[68]
			CD14^+^ (peripheral blood)		

*Abbreviations*: *AKI* acute kidney injury, *BAL* bronchoalveolar lavage, *BM* bone marrow, *CIA* collagen-induced arthritis, *CNS* central nervous system, *COPD* chronic obstructive pulmonary disease, *D-Gal/LPS* d-galactosamine/lipopolysaccharide, *DSS* dextran sulfate sodium, *EAE* experimental autoimmune encephalomyelitis, *EAU* experimental autoimmune uveoretinitis, *FSGS* focal segmental glomerulosclerosis, *HCV* hepatitis C virus, *HDM* house dust mite, *IBD* inflammatory bowel diseases, *ILD* interstitial lung disease, *MS* multiple sclerosis, *PGIA* proteoglycan-induced arthritis, *PH* pulmonary hypertension, *TGF-β* transforming growth factor-β, *TNBS* 2,4,6-trinitrobenzenesulfonic acid

#### Lungs

Although the lungs were long considered to be sterile, they are constantly exposed to microbiota through inhalation or subclinical microaspiration. Far from being sterile, the lungs harbor an abundance of diverse interacting microbiota that regulate lung immunity and homeostasis [19]. In the healthy lung, two macrophage populations work to maintain lung homeostasis: alveolar macrophages and interstitial macrophages. A third population of monocyte-derived macrophages may be recruited during inflammatory responses [20]. MDSCs are critical for negatively regulating immune responses in inflammatory lung diseases.

Arora et al. and Deshane et al. demonstrated that MDSCs suppress the Th2-dominant allergic inflammation in a murine model of asthma [21, 22]. CD11b^+^Gr1^int^F4/80^+^MDSC-like cells accumulate in the lung and suppress the lung DC-mediated reactivation of primed Th2 cells, which is mediated by IL-10 and Arg-1 [21]. The chemokine CCL2 recruits MDSCs into lung tissues in airway inflammation [23]. In humans, high numbers of CD11b^+^CD14^+^CD16^−^HLA-DR^−^ NO-producing myeloid-derived regulatory cells, which are phenotypically similar to MDSCs, were found in the airways of patients with asthma but not in patients with chronic obstructive pulmonary disease (COPD) or in healthy control subjects [24]. These cells suppressed the proliferation of activated autologous CD4^+^T cells.

Patients with COPD have elevated levels of circulating-lineage HLA-DR^−^CD33^+^CD11b^+^ MDSCs [25]. It was recently reported that collagen type 1^+^CD45^dim^CD34^−^CD14^−^CD15^+^ MDSC-like fibrocytes are increased in the lungs and peripheral blood of COPD patients compared to control subjects [26]. The intensity of collagen type 1 staining, which marks MDSC-like fibrocytes, was positively associated with lung function; these cells appeared to play a role in air trapping, predominately in the upper lobes.

We recently reported that MDSCs are expanded in the lungs of SKG mice with interstitial lung disease (ILD) [27]. Other researchers demonstrated that CCR2^+^ M-MDSCs inhibit collagen degradation and promote lung fibrosis by producing transforming growth factor-β1 (TGF-β1) [28].

The number of circulating activated MDSCs was found to be significantly increased in patients with pulmonary hypertension (PH) compared to control subjects, and was correlated with an increase in mean pulmonary artery pressure [29]. However, a direct mechanistic role for MDSCs in pulmonary hypertension and inflammation-associated vascular remodeling has yet to be defined.

Thus, MDSCs in the lungs appear to suppress inflammation in several diseases, including asthma, COPD, ILD, and PH. However, MDSCs may promote lung fibrosis under certain conditions.

#### Joints

A healthy joint has a thin synovial membrane and synovial fluid that does not contain leukocytes. Under arthritic conditions, leukocytes, including both innate and adaptive immune cells, infiltrate the joint cavity and cause proliferation of the synovium [30, 31]. The infiltrating leukocytes include macrophages, DCs, granulocytes, and lymphocytes. Leukocyte infiltration and the secretion of pro-inflammatory cytokines encourage pre-osteoclasts to mature into osteoclasts that erode the bone. The proliferating synovium secretes matrix metalloproteinases (MMPs) and collagenase, leading to the destruction of cartilage.

Egelston et al. identified MDSCs in the joints of mice with proteoglycan-induced arthritis, a model of rheumatoid arthritis (RA) [32]. G-MDSCs obtained from the synovial fluid suppressed DC maturation and T cell proliferation upon co-culture, primarily by producing NO and ROS (which are usually produced by G-MDSCs). Fujii et al. revealed that MDSCs play a crucial role in regulating mouse collagen-induced arthritis (CIA) [33, 34]. MDSCs from the spleens of arthritic mice suppressed the proliferation of CD4^+^ T cells and their differentiation into Th17 cells in vitro. Moreover, the adoptive transfer of spleen-derived MDSCs reduced the severity of arthritis, and depleting MDSCs canceled this effect in vivo. Another report showed similar results in CIA and antigen-induced arthritis models [35]. Nishimura et al. found that tofacitinib, a JAK inhibitor, facilitates the expansion of MDSCs and ameliorates arthritis in SKG mice [36]. On the other hand, Zhang et al. reported that MDSCs are pro-inflammatory and aggravate arthritis in CIA [37]. However, although this group phenotypically defined CD11b^+^Gr1^+^ cells as MDSCs, the cells could not be distinguished from neutrophils or monocytes.

Little is known about MDSCs in human arthritis. Kurkó et al. reported that MDSCs were present in the synovial fluid (SF) of RA patients [38], and that while CD11b^+^CD33^+^HLADR^lo/−^CD14^−^CD15^+^ G-MDSC-like cells were predominant, there was also a small CD11b^+^CD33^+^HLA-DR^lo/−^CD14^+^CD15^−^ monocytic subset. The SF-MDSCs from RA patients significantly suppressed the anti-CD3/CD28 antibody-induced proliferation of autologous T cells.

These findings indicate that MDSCs, especially G-MDSCs, ameliorate arthritis by suppressing T cell proliferation and DC maturation in the joints.

#### Liver

The liver clears gut-derived microbial products from the blood, while functions as a metabolic organ. The unique microenvironments in the liver induce tolerogenic myeloid cells, including MDSCs, under conditions such as liver inflammation and fibrosis [39]. The recruitment and differentiation of MDSCs in the liver is promoted by mechanisms that depend on contact between various cell types and on soluble mediators. For example, hepatic stellate cells can induce MDSCs from myeloid cells in mice and humans [40]. The induction of MDSCs by hepatic stellate cells depends on CD44-mediated, direct cell–cell contact in humans [41], but on soluble factors such as IFN-γ or complement C3 in mice [42, 43]. Human mesenchymal stromal cells can also induce the expansion of MDSCs via hepatocyte growth factor and its receptor, c-Met [43]. IL-6 induces MDSCs to accumulate in the liver, where they protect mice from liver injury mediated by CD8^+^ T cells [44].

The frequency of MDSCs is elevated in the peripheral blood of patients with chronic hepatitis C virus (HCV) infection [45, 46]. CD11b^+^HLA-DR^low^CD14^+^CD33^+^ MDSCs in the blood of patients with chronic HCV suppress T cells using arginase [45]. MDSCs also produce ROS, which inhibit T cell function in chronic HCV [46], and this might be one of the mechanisms by which HCV promotes persistent infection.

MDSCs are involved in the pathogenesis of a mouse model of autoimmune hepatitis. Th1 cell-mediated liver inflammation can induce MDSCs in BALB/c *Tgfb1*^*−/−*^ mice, which develop an acute autoimmune liver injury [47]; IFN-γ production by CD4^+^ T cells is necessary for inducing MDSCs in this model. MDSCs, especially M-MDSCs, were found to suppress CD4^+^ T cell proliferation in this model via NO production, IFN-γ, and cell–cell contact. Zhang et al. also reported that MDSCs are involved in murine immunological hepatic injury [48]. Inhibiting mammalian target of rapamycin (mTOR) with rapamycin induced the recruitment of CD11b^+^Gr1^+^Ly6C^hi^ MDSCs to the liver and protected against immunological hepatic injury. Downregulating the mTOR activity in MDSCs induced iNOS and NO, and the pharmacological inhibition of iNOS completely eliminated the recruitment of MDSCs.

MDSCs were elevated in the liver of a murine fulminant hepatitis model [49]; in this model, IL-25 induced MDSCs to accumulate in the liver, where they protected mice from acute hepatic damage induced by d-galactosamine and lipopolysaccharides.

Some studies indicate that MDSCs are involved in chronic liver injury and the development of liver fibrosis. Suh et al. reported that bone marrow-derived MDSCs ameliorated carbon tetrachloride-induced liver fibrosis in mice by producing IL-10 [50]. Activated hepatic stellate cells enhanced the MDSC expression of IL-10, which in turn suppressed the profibrotic function of activated hepatic stellate cells. Another study showed that M-MDSCs accumulated in the liver in the presence of inflammation and fibrosis in bile duct-ligated mice [51]. In addition, depleting MDSCs in the liver enhanced fibrosis markers, indicating that MDSCs play a protective role against organ fibrosis.

Thus, in the liver, MDSCs (especially M-MDSCs) prevent liver inflammation and fibrosis by suppressing immunogenic T cells.

#### Kidneys

The kidneys are frequently targeted by pathogenic immune responses against renal autoantigens or by local manifestations of systemic autoimmunity [52, 53]. Inflammation and immune system activation are important causal factors in the development of both acute and chronic renal disease [54]. Recent reports indicate that MDSCs are a key regulator for terminating excessive inflammation, but can also contribute to renal fibrosis.

MDSCs, especially G-MDSCs, ameliorate acute kidney injury (AKI). Rapamycin enhanced this protective effect by recruiting MDSCs, regulating MDSC induction, and strengthening the MDSC immunosuppressive activity in a mouse AKI model [55]). In mice fed an adenine-enriched diet, G-MDSCs were found to accumulate in the kidney during chronic kidney inflammation and fibrosis [51]. In addition, depleting MDSCs in the kidney enhanced fibrosis markers, indicating that MDSCs play a protective role against fibrosis. Li et al. reported that the efficacy of glucocorticoids in ameliorating focal segmental glomerulosclerosis (FSGS) depends on the MDSCs’ capacity to expand [56]. After glucocorticoid treatment, the frequency of CD11b^+^HLA-DR^−^CD14^−^CD15^+^ MDSCs in peripheral blood was increased in patients with glucocorticoid-sensitive FSGS. In mice, glucocorticoid treatment increased the frequency of MDSCs in the peripheral blood, bone marrow, spleen, kidney-draining lymph nodes, and kidney. The induced MDSCs from glucocorticoid-treated mice were found to strongly suppress T cells, DCs, and macrophages but to induce regulatory T cells in the spleen, kidney-draining lymph nodes, and kidney.

Thus, MDSCs, especially G-MDSCs, suppress inflammation and fibrosis in the kidney by controlling the excessive expansion of effector T cells, DCs, and macrophages.

#### Intestine

The intestine, which is the largest immune organ, is continually exposed to antigens and immunomodulatory agents from the diet and from commensal microbiota [57]. An imbalance between effector T cells and regulatory cells in the intestine causes mucosal and systemic inflammation. Many studies report relationships between MDSCs and inflammatory bowel diseases (IBD) in humans and experimental mouse model systems [58]. In the first description of MDSC development in a murine IBD model, Haile et al. reported that MDSCs were induced in both the spleen and intestine and that they suppressed CD8^+^ T cell proliferation ex vivo by producing NO [59]. The same study showed that the peripheral blood from IBD patients had an increased frequency of MDSCs, denoted as CD14^+^HLA^−^DR^−/low^ cells. Su et al. showed that the adoptive transfer of bone marrow-derived G-MDSCs into mice with 2,4,6-trinitrobenzenesulfonic acid (TNBS)-induced colitis improved survival in the recipient mice and decreased injury scores, myeloperoxidase activities, and serum IL-6 levels [60]. Other researchers reported that the adoptive transfer of splenic MDSCs reduced inflammation and promoted mucosal healing in a dextran sulfate sodium (DSS)-induced colitis model [61]. MDSCs are induced by a strong variety factors such as granulocyte colony-stimulating factor (G-CSF), GM-CSF, TGF-β, and inflammatory cytokines. In addition to these factors, the antioxidant resveratrol, a naturally occurring polyphenol, induced Arg-1-expressing MDSCs in the colon of IL-10^−/−^ mice [62].

These findings suggest that both M-MDSCs and G-MDSCs protect the intestines from excessive inflammation.

#### Central nervous system

The CNS has long been considered an immune-privileged site because the brain parenchyma, the interstitial fluid, and the cerebrospinal fluid (CSF) are isolated from the blood by two barriers, the blood–brain barrier (BBB) and the blood–CSF barrier (BCSFB). Despite these barriers, immune cells are detected in both the brain parenchyma and the CSF. Under pathological conditions, the integrity of the BBB and the BCSFB can be disturbed to allow the entry of inflammatory cells [63]. Recent studies have shown that, in the context of neuroimmunology, MDSCs are both powerful controllers of T cell activity and important modulatory agents for recovery from immunological insults [64].

Experimental autoimmune encephalomyelitis (EAE) is a commonly used mouse model of multiple sclerosis (MS), a T cell-mediated autoimmune inflammatory disease of the CNS [65]. Several recent studies report that MDSCs are involved in MS. Zhu et al. revealed that CD11b^+^Ly6C^hi^ cells accumulated in the bone marrow, blood, spleen, and CNS of mice during the course of EAE and that M-MDSCs isolated from the spleen suppressed the proliferation of CD4^+^ T cells and CD8^+^ T cells via NO production ex vivo [66]. Interestingly, the regulation of T cells by the CD11b^+^Ly6C^hi^ cells was determined by the MDSCs’ activation state and was plastic during the immune response [67]. IFN-γ, GM-CSF, TNF-α, and CD154 derived from the T cells activated the CD11b^+^Ly6C^hi^ cells during their interactions. Activated CD11b^+^Ly6C^hi^ cells suppressed T cells, but non-activated CD11b^+^Ly6C^hi^ cells functioned as antigen-presenting cells (APCs). In EAE, the CD11b^+^Ly6C^hi^ cells in the CNS were increasingly activated from disease onset to peak, and switched their function from antigen presentation to T cell suppression. Furthermore, the transfer of activated CD11b^+^Ly6C^hi^ cells enhanced T cell apoptosis in the CNS and suppressed EAE. On the other hand, CD11b^+^Ly6G^+^ cells, which are G-MDSCs, accumulate abundantly within the peripheral lymphoid compartments and CNS of mice with EAE prior to remission of the disease [68]. Although the contribution of MDSCs to MS is less clear in humans, Ioannou et al. found significantly more G-MDSCs in the peripheral blood of MS patients at relapse than in the remission period or in control subjects [68]).

M-MDSCs are also critical in resolving acute inflammation and subsequently repairing the damaged tissue after spinal cord injury (SCI) in mice [69]. Furthermore, HMGB1-R AGE signaling promoted the expansion of CD11b^+^Ly-6C^+^MHC-II^low^ cells, which helped to resolve inflammation after a stroke in a murine ischemia model [70]. Interestingly, a similar increase in CD11b^+^CD33^+^HLA-DR^−^ MDSCs was detected in the peripheral blood of stroke patients.

Collectively, both M-MDSCs and G-MDSCs contribute to the resolution of T cell-mediated CNS inflammation and the repair of CNS injury.

#### Eyes

In the eyes, local immune and inflammatory responses are limited, to preserve vision. This phenomenon, known as ocular immune privilege, is mediated by a combination of local and systemic mechanisms [71]. In addition, backup systems to resolve inflammation are needed if ocular immune privilege fails to work. MDSCs have recently emerged as an important cellular component in resolving eye inflammation [72]. Jeong et al. recently reported that HLA-DR^+^CD11b^+^CD33^+^CD14^+^ MDSCs in humans and CD11b^+^Ly6G^−^Ly6C^+^ MDSCs in mice are markedly increased during and before the resolution phase of autoimmune uveoretinitis [73]. In experimental autoimmune uveoretinitis (EAU) mice during remission, the M-MDSCs were increased not only in the spleen and blood, but also in the retina. M-MDSCs isolated from EAU mice suppressed T cell proliferation in vitro, and the adoptive transfer of these cells accelerated the remission of autoimmune uveoretinitis in vivo.

These findings indicate that M-MDSCs serve as negative regulators to resolve eye inflammation.

#### MDSC plasticity

The plasticity of MDSCs has been discussed particularly in tumor models. MDSCs can differentiate into DCs, macrophages, and granulocytes. M-MDSCs from the spleen of EL-4 tumor-bearing mice differentiate into CD11b^+^CD11c^+^ DCs and CD11b^+^F4/80^+^ macrophages with GM-CSF stimulation in vitro [14]. CD11b^+^Gr1^+^ cells isolated from the spleens of tumor-bearing mice differentiate into CD11b^+^Gr1^+^F4/80^+^ suppressor macrophages under the influence of tumor-derived factors in vitro [74]. Furthermore, CD11b^+^Gr1^+^ cells could differentiate into immunostimulatory DCs if cultured with Th1 cytokines, IL-3, and GM-CSF. Hypoxia elicited by hypoxia-inducible factor (HIF)-1α dramatically alters the function of MDSCs in the tumor microenvironment and redirects their differentiation toward tumor-associated macrophages (TAMs) [75]. G-MDSCs cultured in vitro for 24 h in the presence of GM-CSF resemble neutrophils in both phenotype and function [76]. Moreover, MDSCs isolated from a bone-tumor microenvironment differentiate into osteoclasts in the presence of macrophage colony-stimulating factor (M-CSF) and receptor activator or NF-κB ligand (RANKL), and promote the destruction of bone in a mouse model of breast cancer metastasis to the bone [77].

**Fig. 1:**
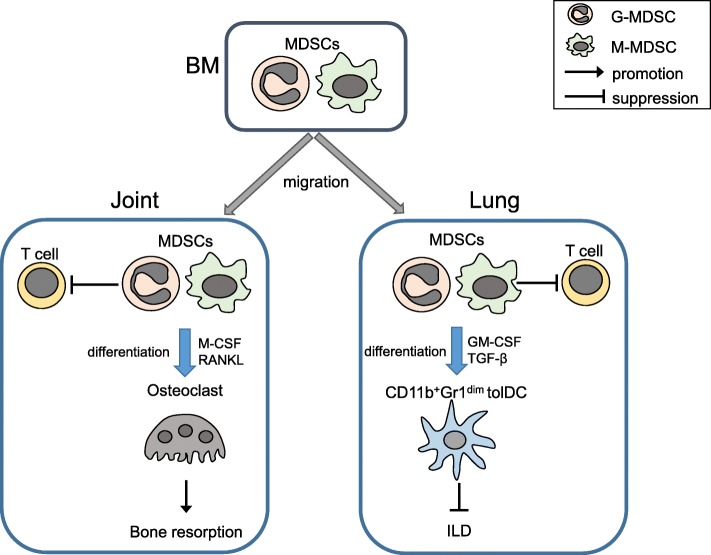
The plasticity of MDSCs in non-neoplastic inflammatory conditions. MDSCs from the bone marrow of CIA mice can differentiate into osteoclasts capable of bone resorption in vitro and in vivo. In the lungs of SKG mice, M-MDSCs differentiate into CD11b^+^Gr1^dim^ tolerogenic DCs (CD11b^+^Gr1^dim^ tolDCs), which suppress the progression of ILD

Although little is known about the plasticity of MDSCs in non-neoplastic inflammatory conditions, recent reports demonstrate that they can differentiate into osteoclasts and tolerogenic DCs (tolDCs) (Fig. 1). Zhang et al. reported that MDSCs from the bone marrow of CIA mice can differentiate into tartrate-resistant acid phosphatase (TRAP)^+^ osteoclasts by the stimulation of M-CSF and RANKL, which are capable of bone resorption in vitro and in vivo [78]. We recently showed that in the lungs of SKG mice, M-MDSCs differentiated into CD11b^+^Gr1^dim^ tolerogenic DCs (CD11b^+^Gr1^dim^ tolDCs), which suppressed the progression of ILD [27]. In that report, intraperitoneally injected zymosan induced ILD with various severity. The MDSC proportion were elevated in lungs with mild or moderate ILD, and the CD11b^+^Gr1^dim^ tolDC population in particular was expanded in the lungs with severe ILD. Th17 cells and groups 1 and 3 innate lymphoid cells (ILC1s and ILC3s), which produced GM-CSF, were elevated in the lungs, and GM-CSF induced the M-MDSCs to differentiate into CD11b^+^Gr1^dim^ tolDCs in vitro. CD11b^+^Gr1^dim^ tolDCs suppressed T cell proliferation in vitro and suppressed the progression of ILD in vivo. Neutralizing TGF-β during the in vitro generation of CD11b^+^Gr1^dim^ tolDCs partially canceled the cells’ suppressive effect on T cell proliferation, indicating that TGF-β was critical for the tolerogenic nature of the CD11b^+^Gr1^dim^ tolDCs. These results together suggested that MDSCs suppress pathogenic lymphocytes in the early stages of inflammation. When inflammation becomes severe, GM-CSF and TGF-β induce M-MDSCs to differentiate into CD11b^+^Gr1^dim^ tolDCs, which further suppress lymphocytes to control excessive inflammation. Our report indicated that MDSCs can differentiate into unique tolDCs in the severely inflamed lung.

These reports indicate that MDSCs could differentiate into both pro-inflammatory and anti-inflammatory cells in the inflamed organs under pathogenic conditions.

## Conclusions

MDSCs accumulate in inflamed organs. IL-6, the chemokine CCL2, rapamycin, and oxidizing agents recruit MDSCs from the bone marrow into the liver, lungs, kidneys, and intestines, respectively. On the other hand, hepatic stellate cells and mesenchymal stromal cells can induce MDSCs from myeloid cells in the liver. MDSCs negatively control inflammation by suppressing T cells and myeloid cells in inflamed organs. Moreover, they regulate fibrosis in the lungs, liver, and kidneys and may be involved in repairing CNS injuries. G-MDSCs act mostly in the joints, kidneys, intestines, and CNS, while M-MDSCs act in the lungs, liver, intestines, CNS, and eyes. MDSCs in organ microenvironments are plastic; MDSCs can differentiate into osteoclasts in the joints, and M-MDSCs can differentiate into tolDCs in the lungs. Further studies are required to characterize the behavior of MDSCs in various organs under non-neoplastic inflammatory conditions.

## Abbreviations

AKI: Acute kidney injury
APC: Antigen-presenting cells
Arg-I: Arginase-I
BBB: Blood–brain barrier
BCSFB: Blood–CSF barrier
CIA: Collagen-induced arthritis
CNS: Central nervous system
COPD: Chronic obstructive pulmonary disease
CSF: Cerebrospinal fluid
DC: Dendritic cell
DSS: Dextran sulfate sodium
EAE: Experimental autoimmune encephalomyelitis
EAU: Experimental autoimmune uveoretinitis
FSGS: Focal segmental glomerulosclerosis
G-CSF: Granulocyte colony-stimulating factor
GM-CSF: Granulocyte macrophage colony-stimulating factor
HCV: Hepatitis C virus
HIF: Hypoxia-inducible factor
IBD: Inflammatory bowel diseases
IFN: Interferon
IL: Interleukin
ILC: Innate lymphoid cell
ILD: Interstitial lung disease
iNOS: Inducible nitric oxide synthase
M-CSF: Macrophage colony-stimulating factor
MDSC: Myeloid-derived suppressor cell
MMP: Matrix metalloproteinase
MS: Multiple sclerosis
mTOR: Mammalian target of rapamycin
NO: Nitric oxide
PBMC: Peripheral blood mononuclear cell
PH: Pulmonary hypertension
RA: Rheumatoid arthritis
RANKL: Receptor activator or NF-κB ligand
ROS: Reactive oxygen species
SCI: Spinal cord injury
SF: Synovial fluid
STAT: Signal transducer and activator of transcription
TAM: Tumor-associated macrophage
TGF-β: Transforming growth factor-β
TNBS: 2,4,6-Trinitrobenzenesulfonic acid
tolDC: Tolerogenic dendritic cell
TRAP: Tartrate-resistant acid phosphatase
Treg: Regulatory T cell
